# Benefit profile of recombinant human soluble thrombomodulin in sepsis-induced DIC

**DOI:** 10.1186/cc13433

**Published:** 2014-03-17

**Authors:** K Yamakawa, H Ogura, S Fujimi, M Morikawa, Y Ogawa, Y Umemura, Y Inoue, H Tanaka, T Hamasaki, T Shimazu

**Affiliations:** 1 Osaka University Graduate School of Medicine, Osaka, Japan; 2 Osaka General Medical Center, Osaka, Japan; 3 Juntendo University Urayasu Hospital, Chiba, Japan

## Introduction

Recombinant human soluble thrombomodulin (rhTM) demonstrated promising evidence suggestive of efficacy in a phase IIb, randomized, controlled trial [1] and is currently under evaluation in a phase III trial. However, the benefit profiles of rhTM have not been elucidated. The purpose of this study was to explore whether patients with a high disease severity (according to Acute Physiology and Chronic Health Evaluation (APACHE) II and Sequential Organ Failure Assessment (SOFA) scores) might have a treatment benefit from rhTM administration.

## Methods

This was a post-hoc, subgroup analysis of a multicenter retrospective cohort study [2] conducted in three tertiary referral hospitals in Japan. All patients with sepsis-induced DIC who required ventilator management were included. We stratified all patients with different disease severity, as defined by APACHE II and SOFA scores to three strata. Intervention effects estimated as hazard ratios were analyzed by Cox regression analysis adjusted for propensity model to detect subgroup heterogeneity of the effects of rhTM on in-hospital mortality.

## Results

**Figure 1:**
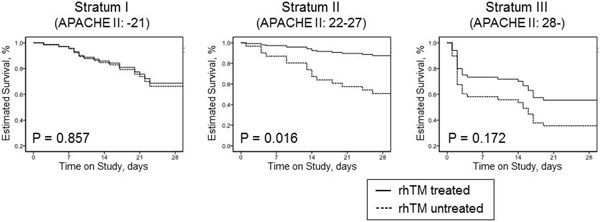
**Estimated survival curves for each of three disease severities**.

Eligible were 162 patients with sepsis-induced DIC; 68 patients received rhTM and 94 did not. After adjusting for imbalances, rhTM administration was significantly associated with reduced mortality only in patents in the stratum II group (APACHE II, 22 to 27) (adjusted hazard ratio, 0.20; 95% confidence interval, 0.05 to 0.74; *P *= 0.016), while not significant in stratum I and stratum III (Figure 1). A similar tendency was observed in analysis for SOFA score (stratum I (SOFA, -10), *P *= 0.368; stratum II (SOFA, 11 to 12), *P *= 0.012; stratum III (SOFA, 13-), *P *= 0.673).

## Conclusion

A survival benefit with rhTM treatment was observed in sepsis-induced DIC and a high risk of death according to baseline APACHE II and SOFA scores.
